# Myocardial infarction causes sex-dependent dysfunction in vagal sensory glutamatergic neurotransmission that is mitigated by 17**β**-estradiol

**DOI:** 10.1172/jci.insight.181042

**Published:** 2024-07-22

**Authors:** Asokan Devarajan, Kerry Wang, Zulfiqar A. Lokhandwala, Maryam Emamimeybodi, Kassandra Shannon, John D. Tompkins, Andrea L. Hevener, Aldons J. Lusis, E. Dale Abel, Marmar Vaseghi

**Affiliations:** 1 Division of Cardiology,; 2 Department of Medicine,; 3 Division of Endocrinology, Diabetes, and Hypertension,; 4 Department of Microbiology, Immunology, and Molecular Genetics, and; 5 Molecular, Cellular, and Integrative Physiology Interdepartmental Program, UCLA, Los Angeles, California, USA.

**Keywords:** Cardiology, Arrhythmias, Cardiovascular disease, Innervation

## Abstract

Parasympathetic dysfunction after chronic myocardial infarction (MI) is known to predispose ventricular tachyarrhythmias (ventricular tachycardia/ventricular fibrillation [VT/VF]). VT/VF after MI is more common in males than females. The mechanisms underlying the decreased vagal tone and the associated sex difference in the occurrence of VT/VF after MI remain elusive. In this study, using optogenetic approaches, we found that responses of glutamatergic vagal afferent neurons were impaired following chronic MI in male mice, leading to reduced reflex efferent parasympathetic function. Molecular analyses of vagal ganglia demonstrated reduced glutamate levels, accompanied by decreased mitochondrial function and impaired redox status in infarcted males versus sham animals. Interestingly, infarcted females demonstrated reduced vagal sensory impairment, associated with greater vagal ganglia glutamate levels and decreased vagal mitochondrial dysfunction and oxidative stress compared with infarcted males. Treatment with 17β-estradiol mitigated this pathological remodeling and improved vagal neurotransmission in infarcted male mice. These data suggest that a decrease in efferent vagal tone following MI results from reduced glutamatergic afferent vagal signaling that may be due to impaired redox homeostasis in the vagal ganglia, which subsequently leads to pathological remodeling in a sex-dependent manner. Importantly, estrogen prevents pathological remodeling and improves parasympathetic function following MI.

## Abstract 2

Myocardial infarction leads to dysfunction of sensory neurons of the vagal ganglia, with associated mitochondrial dysfunction and oxidative stress.

## Graphical abstract

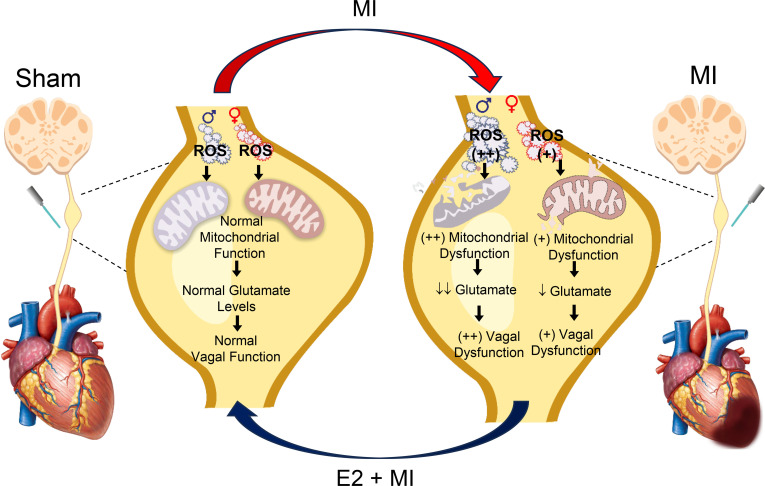

## Introduction

Myocardial infarction (MI) leads to heart failure and sudden cardiac death (SCD). Ventricular arrhythmias, such as ventricular tachycardia (VT) and ventricular fibrillation (VF), remain an important cause of SCD after MI (1, 2). Sex differences have been reported in the incidence of SCD, with a lower incidence noted in women (3). Men with structural heart disease have a higher incidence of VT/VF in the setting of both ischemic and nonischemic cardiomyopathy (4, 5). It has also been observed that women with coronary artery disease have a lower risk of recurrent MI and coronary events, as well as all-cause mortality, than men (6). Furthermore, the incidence of ventricular arrhythmias and appropriate implantable cardioverter-defibrillator (ICD) shocks have been reported to be lower in women than men (7–9), and it has been suggested that this difference may be related to the occurrence of arrhythmia triggers or hormonal differences (10, 11), though underlying mechanisms remain unknown.

The cardiac autonomic nervous system, which includes the sympathetic and parasympathetic branches, controls every aspect of cardiac function (12). Cardiovascular disease causes significant sympathovagal imbalance, characterized by sympathetic activation and parasympathetic dysfunction (vagal withdrawal) (13, 14). This cardiac autonomic remodeling plays an important role in the genesis of VT/VF and the progression of heart failure, increasing the risk of SCD and mortality (13, 14). Sympathetic activation and vagal dysfunction work in concert to increase arrhythmia triggers and increase electrophysiological heterogeneity that predisposes to the occurrence of ventricular arrhythmias (14). Importantly, patients with decreased central vagal tone, as reflected by decreased heart rate (HR) variability and baroreflex sensitivity (BRS), have a significantly increased risk of VT/VF and SCD (15–18).

While many of the mechanisms leading to sympathoexcitation have been well characterized, the mechanisms underlying parasympathetic dysfunction and decreased vagal tone remain unclear. Prior studies have shown that activation of cardiac afferent vagal sensory neurons residing in the inferior vagal (nodose) ganglia reflexively increases vagal outflow to the heart under healthy physiological conditions, resulting in increased vagal efferent tone, which leads to decreases in HR and blood pressure (BP) (19–21). Hence, it is possible that remodeling in sensory afferent neurons of the vagal ganglia could contribute to decreased efferent vagal tone. How these afferent pathways remodel after MI remains poorly characterized. In addition, sensory/afferent vagal neurotransmission depends on glutamatergic afferent neurons that, in turn, depend on healthy mitochondria to produce glutamate (22, 23). A number of preclinical and clinical studies have demonstrated increased generation of reactive oxygen species (ROS) in infarcted myocardial tissue that can affect myocardial mitochondrial function (24). Whether neuronal mitochondrial dysfunction occurs in afferent vagal ganglia neurons after MI and whether this might contribute to the impaired vagal reflexes observed after MI remains unknown. Finally, it is unknown if MI can cause a sex-dependent change in vagal remodeling that may underly the observed differences in propensity to VT/VF observed in females.

In this study, we hypothesized that the reduction in efferent vagal tone after MI is due to dysfunction of afferent neural glutamatergic signaling. We further hypothesized that this impaired sensory neurotransmission may result from pathological vagal glutamatergic remodeling after MI, which may be a consequence of increased oxidative stress and impaired mitochondrial function. Furthermore, we postulated that the sex differences observed in the incidence of ventricular arrhythmias/SCD after MI may be, in part, due to sex differences in the hormonal milieu that modulate pathological vagal neural remodeling, and we specifically examined potential contributions of estrogen.

## Results

### BRS testing in mice after MI.

**Figure 1:**
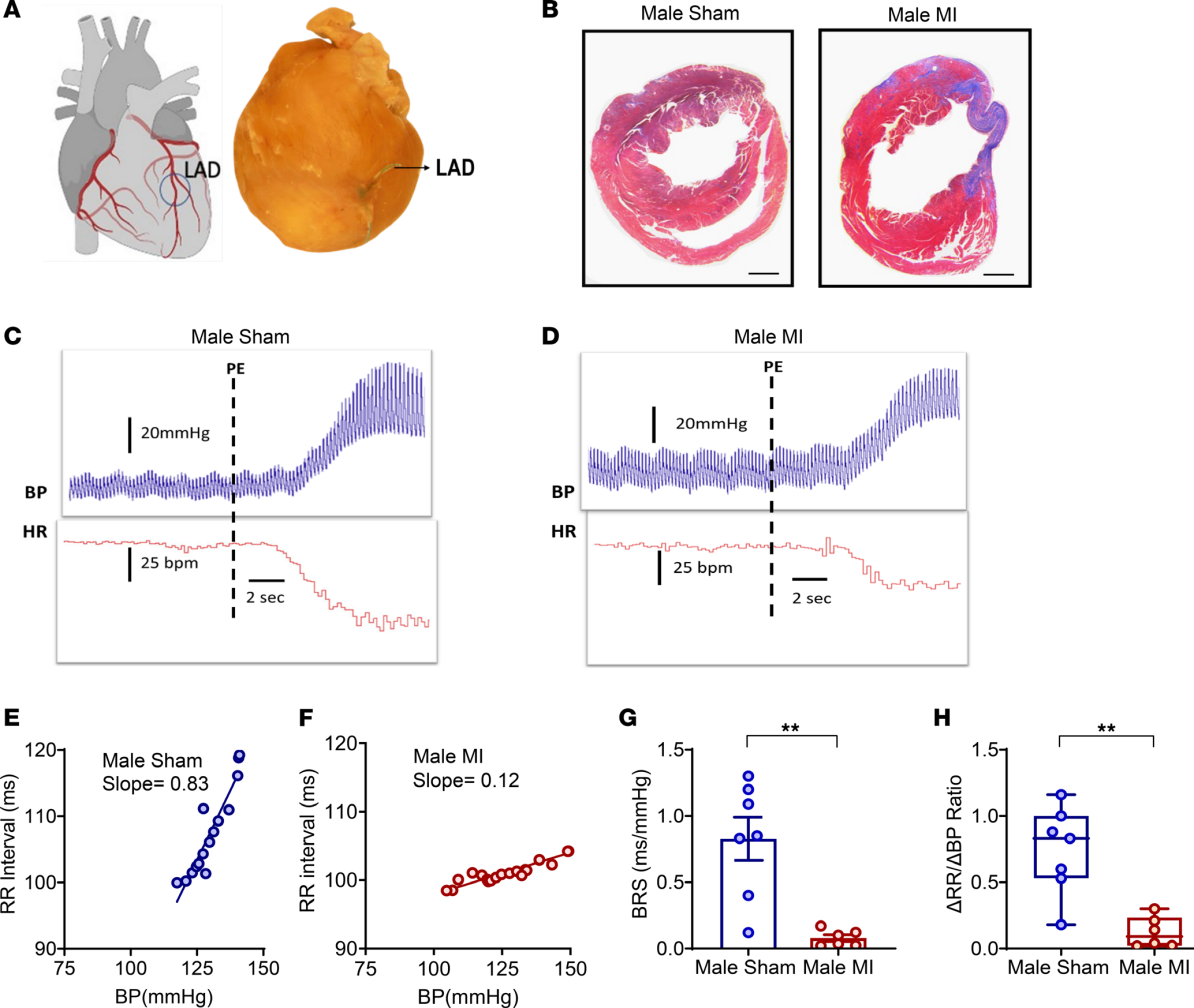
Baroreflex testing in sham and infarcted male mice. (**A**) Myocardial infarction was created by ligation of the LAD coronary artery. (**B**) Two to 3 weeks after MI, the presence of cardiac fibrosis was confirmed in infarcted males. Scale bar: 500 μm. (**C** and **D**) Examples of blood pressure and heart rate tracings after infusion of phenylephrine in male sham and MI mice. (**E** and **F**) BRS was measured as the slope of the beat-to-beat RR interval with respect to the systolic blood pressure. Representative slopes of BRS in a sham and an infarcted male mouse in response to phenylephrine infusion are shown. (**G**) BRS was significantly reduced in male infarcted mice (*n* = 6) versus sham (*n* = 7) mice (*P* < 0.001). (**H**) The ratio of ΔRR/ΔBP was also reduced in male MI versus male sham mice in response to phenylephrine, indicative of vagal dysfunction (*P* < 0.001). Data are shown as mean ± SEM. **P < 0.01; unpaired Student’s *t* test was used for intergroup comparisons.

The arterial baroreflex is critical for the regulation of BP and HR. Rises in BP led to activation of sensory nerve endings of the aortic and carotid baroreceptor neurons that reside in the vagal ganglia. Upon activation of these receptors, afferent signals are transmitted to the nucleus tractus solitarius, ultimately resulting in decreased sympathetic tone and increased cardiac vagal outflow, leading to decreased HR and BP (20, 25). Vagal BRS has been reported to be reduced in patients after MI, and decreased BRS is associated with an increased risk of ventricular arrhythmias and mortality (15, 17, 26, 27). Hence, we first determined whether vagal BRS was reduced in chronically infarcted male mice. MI was created using left anterior descending (LAD) occlusion (Figure 1A), while sham animals underwent thoracotomy only. Cardiac fibrosis was confirmed in infarcted mice using Masson’s trichrome staining (Figure 1B). BRS was calculated as the slope of the linear regression of the beat-to-beat RR interval (inverse of heat rate in msec) versus systolic BP, after infusion of phenylephrine (Figure 1, C–F), 2–3 weeks after MI or sham procedures. BRS was significantly lower in chronic MI compared with sham males (MI [0.078 ± 0.025 ms/mmHg (*n* = 6)] versus sham [0.82 ± 0.16 ms/mmHg (*n* = 7)], *P* < 0.01; Figure 1G). In addition, the ratio of the change in the RR interval to the change in BP (ΔRR/ΔBP) (28) was also lower in infarcted males by nearly 85% (MI [0.12 ± 0.04 (*n* = 6)] versus sham [0.74 ± 0.12 ms/mmHg (*n* = 7)], *P* < 0.01; Figure 1H), indicating the presence of significant vagal baroreflex dysfunction.

**Figure 2:**
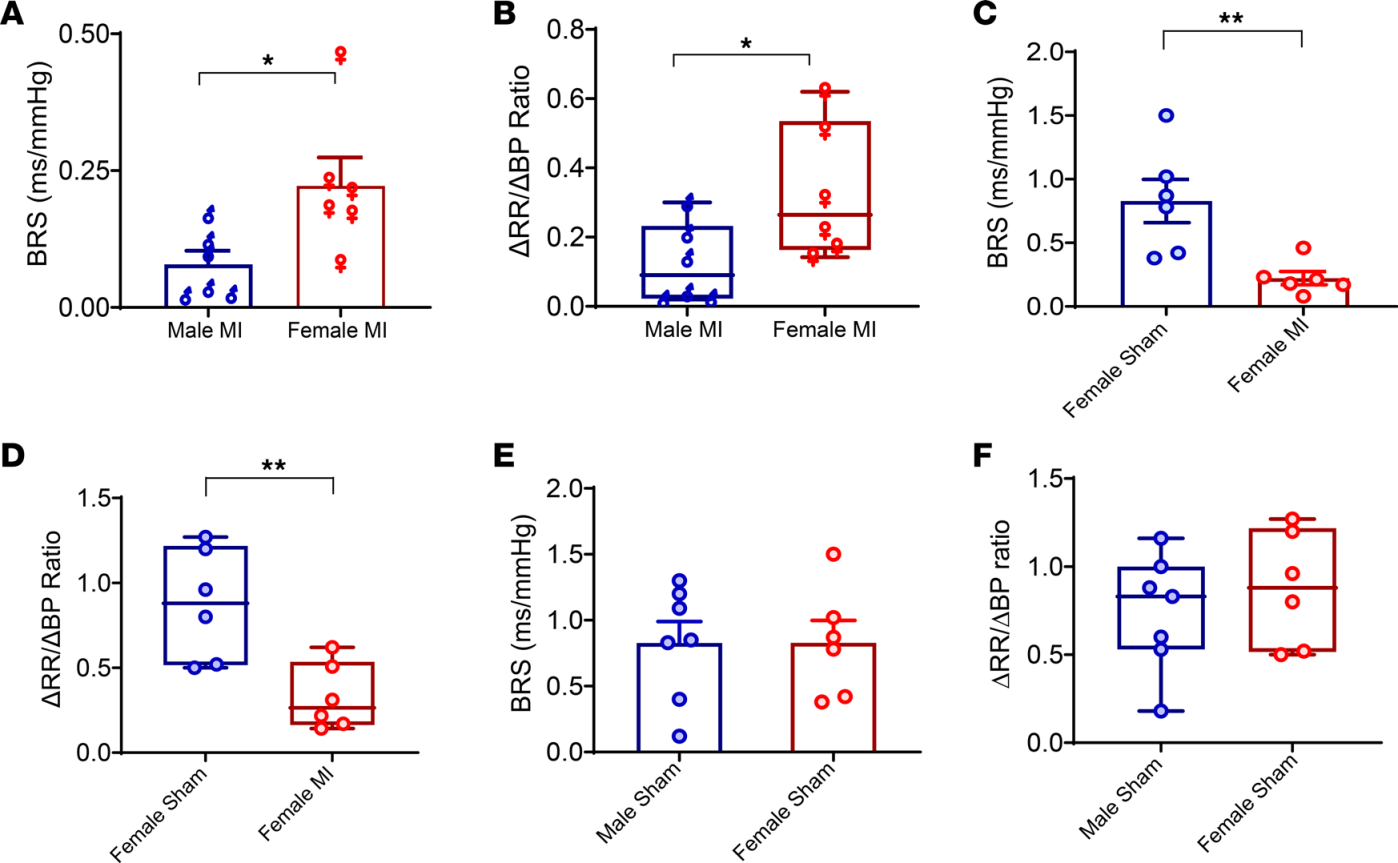
Sex differences in baroreflex sensitivity. (**A**) Infarcted males had lower BRS than infarcted females (*n* = 6 per group, *P* < 0.05). (**B**) The ratio of ΔRR/ΔBP, another measure of baroreflex function, was also found to be lower in infarcted males than females (*P* < 0.05). (**C** and **D**) BRS and the ratio of ΔRR/ΔBP was also blunted in infarcted females (*n* = 6) versus sham (*n* = 6) females. (**E** and **F**) There was no differences in BRS or in ΔRR/ΔBP in male sham (*n* = 7) versus female sham (*n* = 6) animals. Unpaired Student’s *t* test was used for male versus female comparisons. Data are shown as mean ± SEM. *P < 0.05, **P < 0.01 for male MI versus female MI.

Data regarding changes in autonomic function after MI in males versus females are lacking and could explain the sex differences in the observed occurrence of ventricular arrhythmias. Hence, we examined whether BRS after chronic MI differed in male versus female mice. BRS was significantly better in infarcted female versus male mice (female MI [0.2 ± 0.04 ms/mmHg (*n* = 6) ]versus male MI [0.07 ± 0.03 ms/mmHg (*n* = 6)], *P* < 0.05 or males versus females; Figure 2A). ΔRR/ΔBP was also notably greater in infarcted females versus infarcted males (female MI [0.3 ± 0.06 ms/mmHg (*n* = 6)] versus male MI [0.12 ± 0.47 ms/mmHg (*n* = 6)], *P* < 0.05), indicative of significantly greater autonomic dysfunction in male mice (Figure 2B). Compared with female shams, female MI still demonstrated a lower BRS (female sham [0.82 ± 0.16 ms/mmHg (*n* = 6)] versus female MI [0.2 ± 0.04 ms/mmHg (*n* = 6)], *P* < 0.01) and also ΔRR/ΔBP (female sham [0.87 ± 0.13 ms/mmHg (*n* = 6)] versus female MI [0.32 ± 0.06 ms/mmHg (*n* = 6)], *P* < 0.01; Figure 2, C and D), suggesting that MI does still cause some degree of neural remodeling in females. Neither BRS nor ΔRR/ΔBP were different in male sham versus female sham mice (Figure 2, E and F).

### Optical stimulation of glutamatergic neurons in sham and infarcted male mice.

**Figure 3:**
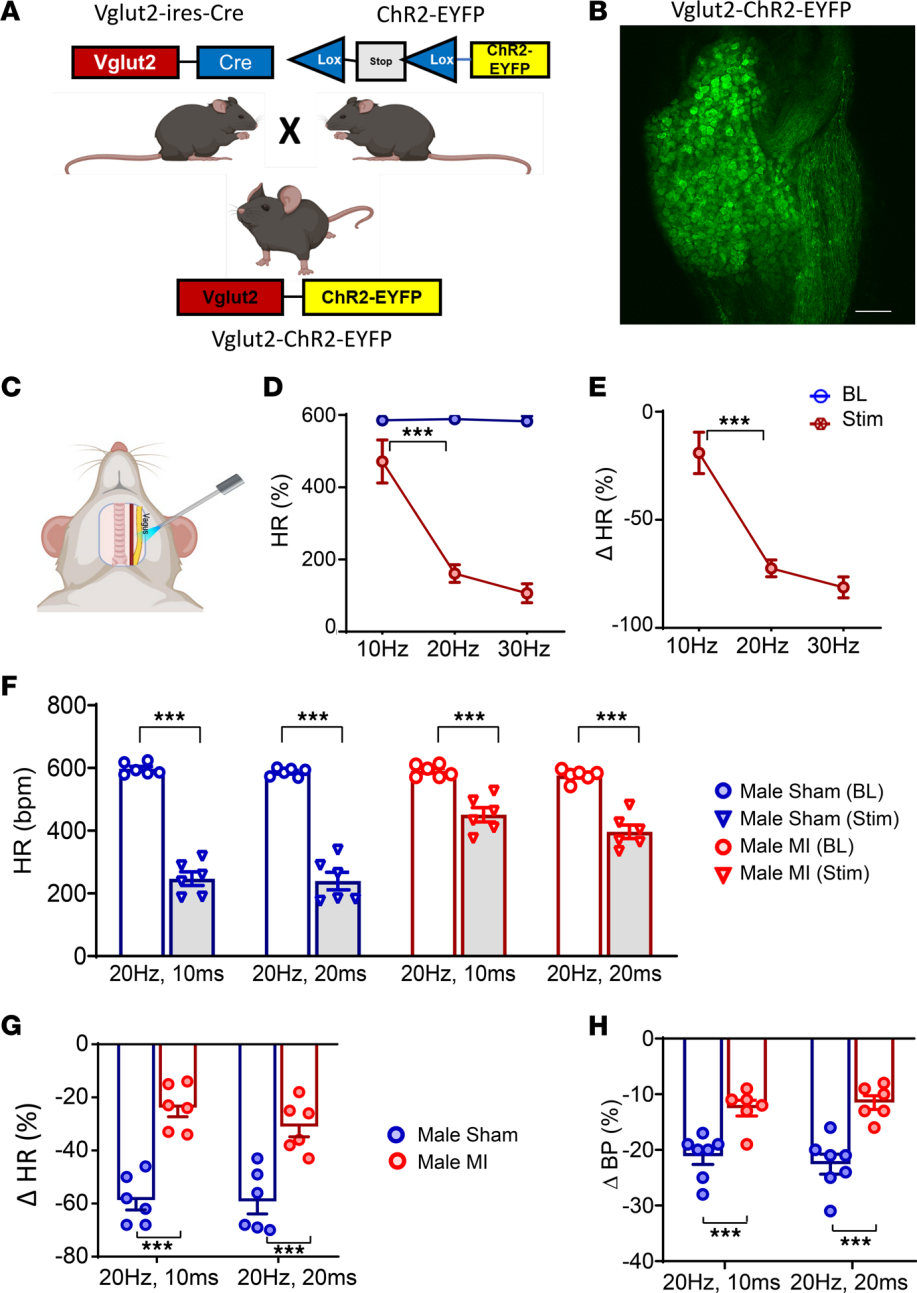
In vivo optogenetic stimulation of vagal sensory neurons in healthy control, sham, and infarcted male mice. (**A** and **B**) Vglut2-ires-Cre mice were crossed with ChR2-EYFP mice to obtain Vglut2-ChR2-EYFP offspring, which was confirmed via genotype testing. In addition, presence of EYFP in the nodose ganglia of Vglut2-ChR2-EYFP mice was also confirmed via confocal microscopy. Scale bar: 50 µm. (**C**–**E**) In vivo responses to left vagal optogenetic stimulation in Vglut2-ChR2-EYFP mice are shown (*n* = 3). (**F**) Two to 3 weeks after MI, in vivo optogenetic stimulation of the left vagus nerve was performed, and hemodynamic responses were quantified at baseline and during stimulation. Both sham (*n* = 6) and infarcted male mice (*n* = 6) showed a decrease in HR in response to optogenetic stimulation (*P* < 0.0001). (**G**) However, changes in HR were greater in sham versus MI animals (20 Hz, 10 ms: male sham versus male MI, *P* < 0.0001; 10 Hz, 20 ms: male sham versus male MI, *P* < 0.0001). (**H**) Similarly, decreases in blood pressure to stimulation were greater in male sham versus MI animals (20 Hz, 10 ms: male sham (*n* = 7) versus male MI (*n* = 6), *P* < 0.0001; 20 Hz, 20 ms: male sham versus male MI, *P* < 0.0001). Data are shown as mean ± SEM. ***P < 0.001. BL, baseline prior to stimulation; Stim, during optical stimulation. Unpaired Student’s *t* test used for comparisons of MI versus sham.

To selectively activate vagal glutamatergic afferent neurons and determine whether vagal sensory dysfunction contributed to the decreased efferent vagal tone observed after MI, Vglut2-ChR2-EYFP mice were used. The presence of ChR2-EYFP was confirmed via genotyping and confocal imaging showing the expression of EYFP in vagal neurons (Figure 3, A and B). The lack of EYFP expression in the vagal ganglia of Vglut2-ires-Cre or ChR2-EYFP mice was also confirmed (Supplemental Figure 1, A and B; supplemental material available online with this article; https://doi.org/10.1172/jci.insight.181042DS1). Initially, dose responses to optogenetic stimulation of the left vagal ganglia in vivo were evaluated at 10, 20, and 30 Hz using a blue light laser (473 nm wavelength) in healthy Vglut2-ChR2-EYFP mice, with Vglut2-ires-Cre and ChR2-EYFP mice used as controls (Figure 3, C–E). Optical stimulation of the vagus nerve in Vglut2-ChR2-EYFP mice resulted in a decrease in HR (10 Hz: from 585 ± 9 bpm to 471 ± 60 bpm; 20 Hz: from 588 ± 5.9 bpm to 161 ± 25 bpm; 30 Hz: from 582 ± 14 bpm to 106 ± 26 bpm; *P* < 0.01 for change from baseline for all frequencies) (Figure 3D), while no changes in HR were observed with optical stimulation of the vagus nerve in Vglut2-ires-Cre or ChR2-EYFP mice (Supplemental Figure 1, C and D). An increase in the frequency of optical stimulation resulted in further decreases in HR in Vglut2-ChR2-EYFP mice (Figure 3, D and E), while HR responses were unchanged regardless of the stimulation frequency in Vglut2-ires-Cre and ChR2-EYFP mice (Supplemental Figure 1, C and D). Given that HR responses to optogenetic stimulation plateaued at ≥ 20 Hz, this frequency was selected for subsequent studies in infarcted and sham animals. As firing frequency increases with pulse width, a pulse width of 10 ms (in addition to 20 ms) was also selected and tested in sham and infarcted animals.

Male Vglut2-ChR2-EYFP underwent MI or sham procedures as described above. Two to 3 weeks after MI or sham procedures, the left cervical vagus nerve was optically stimulated in vivo (20 Hz, 10 and 20 ms pulse widths), and HR and BP responses were assessed. Vagal optical stimulation in Vglut2-ChR2-EYFP infarcted male mice demonstrated significantly attenuated HR responses compared with sham males at both 20 Hz, 10 ms (MI: from 590 ± 7 bpm to 451 ± 21 bpm, *P* < 0.0001; sham: 597 ± 6 bpm to 247 ± 17 bpm, *P* < 0.0001) and 20 Hz, 20 ms of stimulation (MI: from 576 ± 7 bpm to 396 ± 20 bpm, *P* < 0.0001; sham: 587 ± 4 bpm to 240 ± 22 bpm, *n* = 6 per group, *P* < 0.00001) (Figure 3F). Hence, the percentage change in HR to optical stimulation was significantly greater in sham versus infarcted animals (20 Hz, 10 ms: sham –59% ± 3% versus MI –24% ± 3%, *P* < 0.0001; 20 Hz, 20 ms: sham –59% ± 4% versus MI –31% ± 4%, *P* < 0.0001; Figure 3G). Similar to the observed HR responses, the percentage change in BP from baseline in response to optical stimulation was also impaired in infarcted Vglut2-ChR2-EYFP male mice (Figure 3H and Supplemental Figure 2).

**Figure 4:**
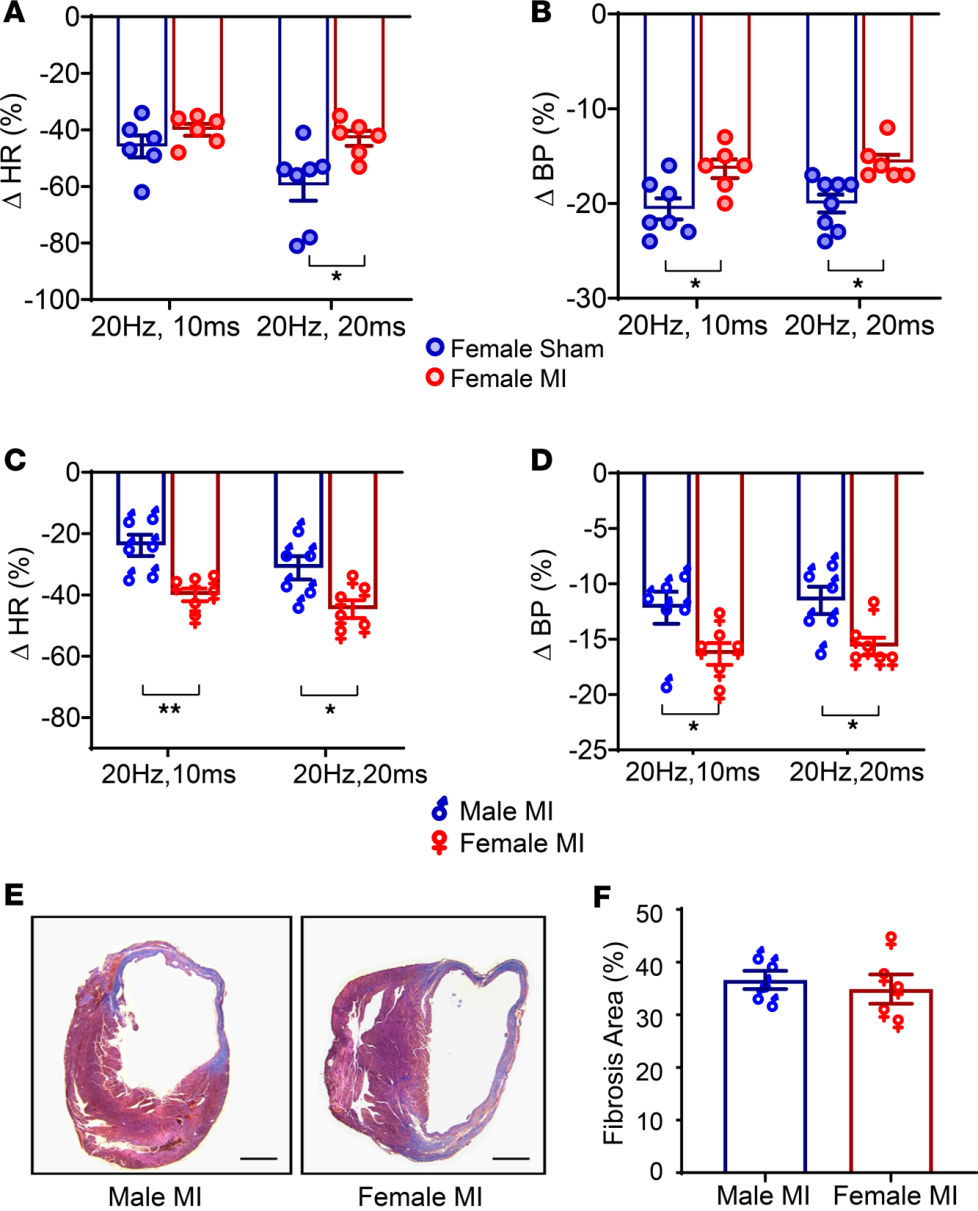
Sex differences in in vivo optogenetic responses. (**A** and **B**) In response to in vivo optogenetic vagal stimulation, female MI animals demonstrated a blunted HR and BP response compared with female sham animals (*n* = 6 per group). (**C**) However, changes in HR were significantly different in male MI versus female MI animals (*n* = 6 per group) at both stimulation parameters (*P* < 0.01), with male animals demonstrating more diminished responses. (**D**) Similar to HR, blood pressure responses to optogenetic stimulation were also reduced in infarcted males versus infarcted females (male MI versus female MI change in blood pressure at 20 Hz, 10 ms: *P* < 0.05; change in blood pressure at 20 Hz, 20 ms: *P* < 0.05, *n* = 6 per group). (**E** and **F**) Myocardial fibrosis was quantified using Masson’s trichrome staining. Scale bar: 500 μm. No difference in the degree of fibrosis between male MI and female MI mice was observed (*n* = 5 per group). Data are shown as mean ± SEM. *P < 0.05, **P < 0.01. BL, baseline (prestimulation); Stim, during optical stimulation. Unpaired Student’s *t* test used for comparisons of males versus females.

Since BRS was reduced in males relative to females after MI, we tested whether underlying sex differences in vagal afferent glutamatergic signaling could cause the observed changes. To this end, the left cervical vagus was optically stimulated in Vglut2-ChR2-EYFP female infarcted and sham animals. HR and BP responses were blunted in female infarcted compared with female sham mice (Figure 4, A and B). However, the percentage change in HR was significantly greater in infarcted females versus infarcted males at 20 Hz, 10 ms (male MI [–24% ± 3% ( *n* = 6)] versus female MI [–40% ± 1.3 % (*n* = 6)]; *P* < 0.001) and at 20 Hz, 20 ms (male MI [–31% ± 3% (*n* = 8)] versus female MI [–45% ± 2% (*n* = 6)], *P* < 0.05; Figure 4C), suggesting significantly greater sensory neural remodeling in infarcted males. Consistent with decreased reflex HR responses, the percentage change in BP was also greater in infarcted females versus infarcted males (Figure 4D). The degree of ventricular fibrosis after MI did not significantly differ between the sexes (Figure 4, E and F), suggesting that the sex differences in autonomic dysfunction could not be attributed to infarct size. Of note, there was no difference in HR or BP responses in male sham versus female sham mice (Supplemental Figure 3).

### Effect of MI on vesicular glutamate transporter and glutamate levels in the vagal ganglia.

**Figure 5:**
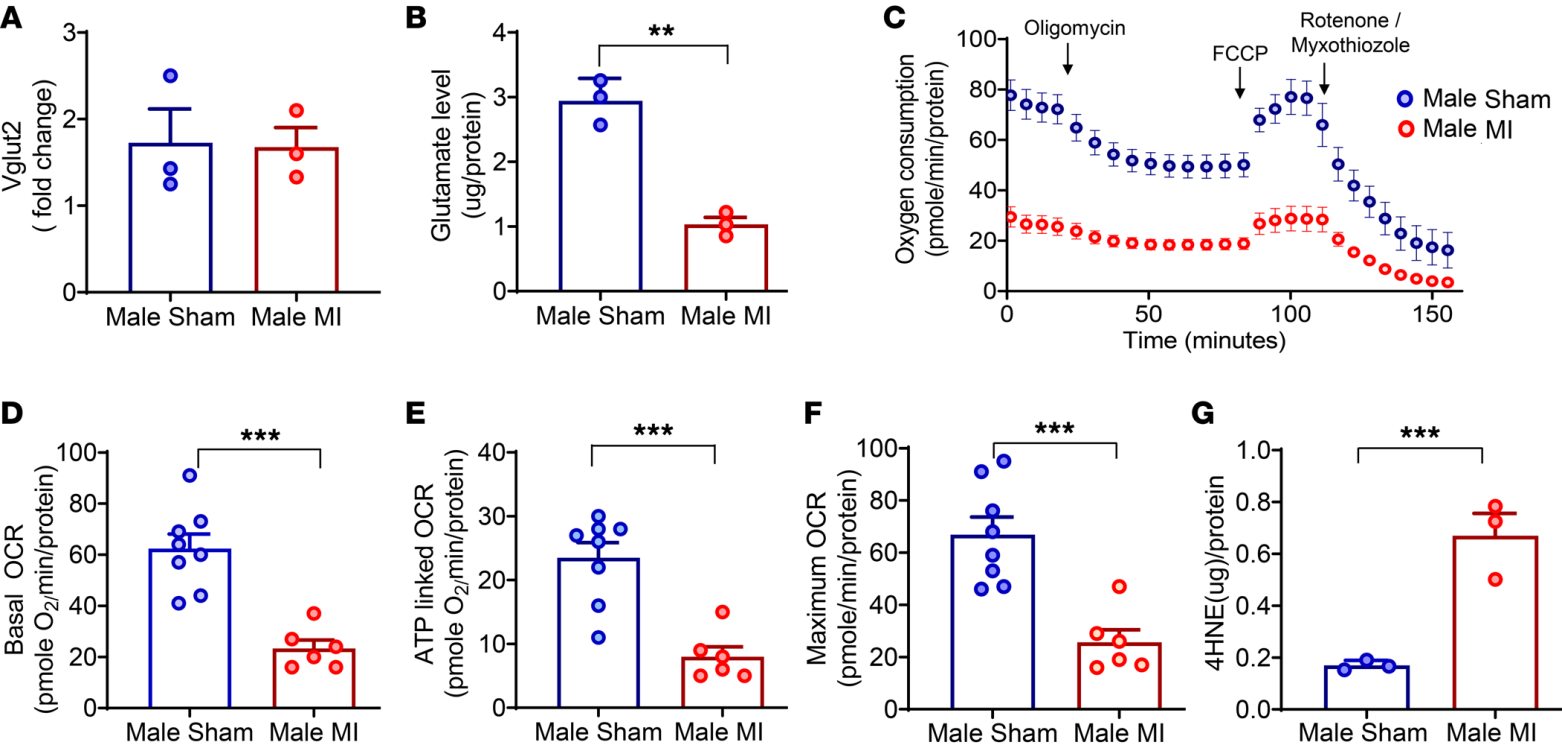
Vglut2 and glutamate levels, mitochondrial function, and oxidative stress in the vagal ganglia of male sham and infarcted mice. (**A** and **B**) Vagal ganglia Vglut2 (*n* = 6 animals/sample, 3 separate samples per group) and glutamate levels (*n* = 9 animals/sample, 3 separate samples per group) were measured. While there was no change in Vglut2 levels, significantly lower glutamate levels (*P* < 0.01) were found in infarcted compared with sham males. (**C**) Mitochondrial oxygen consumption rates (OCRs) were measured in the vagal ganglia of sham (*n* = 8 ganglia) and infarcted (*n* = 6 ganglia) males. (**D**) Basal OCR was lower in infarcted versus sham males (*P* < 0.001). (**E**) ATP-linked OCR was also lower in infarcted versus sham males (*P* < 0.001). (**F**) Maximal OCR, deduced from treatment with FCCP (uncoupler), was found to be lower in infarcted versus sham males (*P* < 0.001). (**G**) 4-HNE levels were significantly lower in infarcted versus sham males (*n* = 6 animals/sample, 3 independent samples per group, *P* < 0.001). Data are shown as mean ± SEM. **P < 0.01, ***P < 0.001. Unpaired Student’s *t* test was used for intergroup comparisons.

To better delineate factors underlying the reduced afferent responses of glutamatergic neurons, we assessed both Vglut2 levels (Vglut2 is responsible for transporting cytoplasmic glutamate into synaptic vesicles) (29) and glutamate levels in the vagal ganglia of infarcted versus sham male mice. Three different types of transporters, Vglut1, Vglut2, and Vglut3, have been reported (30), and previous studies have demonstrated the presence of Vglut2 in the vagal ganglia (31). Hence, we assessed whether a decrease in Vglut2 (leading to decreased glutamate transport into vesicles and decreased neurotransmission) could underly the reduced afferent responses after MI. Assessment of Vglut2 levels in the vagal ganglia of male sham versus infarcted Vglut2-ChR2-EYFP mice demonstrated no significant differences, suggesting that the levels of this transporter remained intact after MI (Figure 5A). We then assessed glutamate content in male sham and infarcted mice to determine if a reduction in the availability of glutamate could underly the differences in sensory neurotransmission observed in these animals. Glutamate levels in the vagal ganglia were significantly decreased after chronic MI in male mice (MI [1.03 ± 0.1 μg/protein] versus sham [2.9 ± 0.2 μg/protein], *P* < 0.01; Figure 5B), suggesting that lower neurotransmitter levels may underly the vagal afferent dysfunction observed.

**Figure 6:**
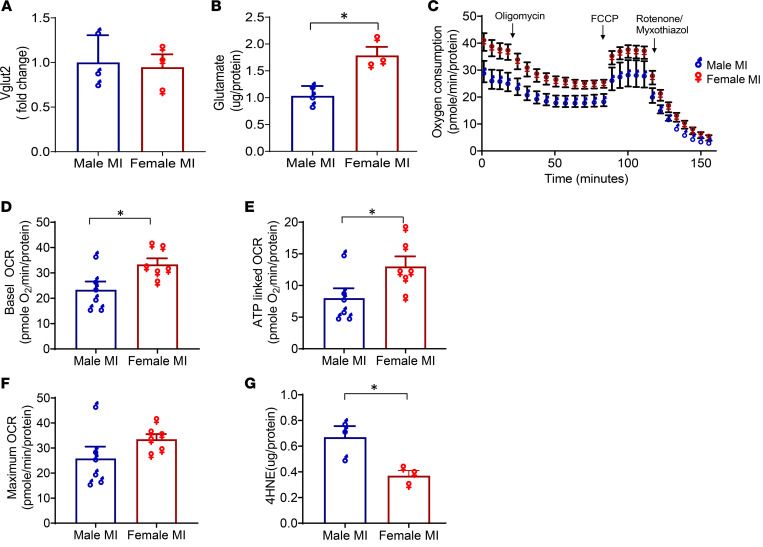
Evaluation of sex differences in Vglut2, glutamate, mitochondrial function, and oxidative stress in the vagal ganglia of male and female mice after MI. (**A** and **B**) Vglut2 (*n* = 6 animals/sample per group, 3 separate samples/experiments) and glutamate levels (*n* = 9 animals/sample per group, 3 separate samples/experiments) were measured from pooled vagal ganglia of infarcted male and infarcted female Vglut2-ChR2-EYFP mice. Vglut2 levels were unchanged, but glutamate levels were significantly reduced in males versus females after MI (*P* < 0.05). (**C**) Oxygen consumption was assessed in the isolated vagal ganglia of Vglut2-ChR2-EYFP males (*n* = 6 ganglia) and females (*n* = 6 ganglia) after MI. (**D**–**F**) Basal mitochondrial (*P* < 0.05), ATP-linked (*P* < 0.05), and FCCP-induced oxygen consumption rates were lower (*P* = 0.05) in males compared with female infarcted mice. (**G**) 4-HNE levels were quantified in isolated vagal ganglia from infarcted Vglut2-ChR2-EYFP male and female mice (*n* = 6 [i.e., 12 ganglia] per sex per sample, 3 independent experiments) and were significantly lower in male compared with female mice (*P* < 0.05). Unpaired Student’s *t* test was used for male versus female comparisons. Data are shown as mean ± SEM. *P < 0.05.

Given the notable sex differences in autonomic dysfunction and afferent glutamatergic neurotransmission after MI, we then assessed whether these differences could be due to either Vglut2 or glutamate levels. While there was no change in Vglut2 levels (Figure 6A), glutamate content was significantly higher in the vagal ganglia of infarcted females versus males (female MI [1.8 ± 0.2 μg/protein] versus male MI [1.0 ± 0.1 μg/protein], *P* < 0.05 for female MI versus male MI; Figure 6B).

### Assessment of mitochondrial function and lipid oxidation after MI.

Recent studies have demonstrated an important link between citric acid cycle intermediates and the regulation of neural pathways, including the synthesis of neurotransmitters and their precursors (22, 32). The regulation and amphibolic nature of the citric acid cycle are dependent on the mitochondrial electron transport chain (oxidative phosphorylation), a process critical to maintaining cell homeostasis (33). Hence, we evaluated whether altered glutamate neurotransmitter levels in sham versus MI Vglut2-ChR2-EYFP male ganglia correlated with mitochondrial dysfunction. To this end, mitochondrial oxygen consumption rates (OCR) in the vagal ganglia were evaluated using a Seahorse extracellular flux analyzer. Vagal ganglia from infarcted male mice showed significantly lower levels of basal mitochondrial OCR than sham mice (MI [21 ± 3 (*n* = 6 ganglia)] versus sham [63 ± 4 pmole O_2_ min/protein (*n* = 8 ganglia)], *P* < 0.001; Figure 5, C and D), suggesting evidence of neuronal mitochondrial dysfunction after MI. Suppression of respiration to oligomycin was reduced in infarcted male mice, suggesting a defect in ATP synthesis (MI [6 ± 0.7 pmole O_2_ min/protein (*n* = 6 ganglia)] versus sham [21.4 ± 1.5 pmole O_2_ min/protein (*n* = 8 ganglia)], *P* < 0.001; Figure 5E). Moreover, maximal rates of mitochondrial respiration following the addition of carbonyl cyanide-p-trifluoromethoxyphenylhydrazone (FCCP) revealed substantially lower OCR in the ganglia of male MI versus sham Vglut2-ChR2-EYFP mice (MI [26 ± 4 pmole O_2_ min/protein (*n* = 6 ganglia)] versus sham [67 ± 4.7 pmole O_2_ min/protein (*n* = 8 ganglia)], *P* < 0.0001 for MI versus sham; Figure 5F). Along with the decrease in mitochondrial OCR, levels of 4-hydroxy-nonelal (4-HNE) — a marker of increased oxidative stress — were significantly higher in the vagal ganglia of infarcted male mice (MI [0.67 ± 0.08 μg/protein] versus sham [0.17 ± 0.01μg/protein], *P* < 0.001; Figure 5G).

To evaluate if lower mitochondrial OCR underlies the sex differences observed in glutamate levels, we then compared OCR levels in infarcted females versus male mice (basal OCR: female MI [33 ± 2 pmole O_2_ min/protein] versus male MI [21 ± 3 pmole O_2_ min/protein], *P* < 0.05; ATP-linked OCR: female MI [13 ± 1 pmole O_2_ min/protein] versus male MI [6 ± 0.8 pmole O_2_ min/protein], *P* < 0.05; FCCP induced OCR: female MI [33 ± 2 pmole O_2_ min/protein] versus male MI [26 ± 5 pmole O_2_ min/protein], *P* = 0.16, *n* = 6 male ganglia and *n* = 6 female ganglia; Figure 6, C–F). Furthermore, levels of the oxidative stress marker 4-HNE were also significantly greater in the vagal ganglia of infarcted males compared with infarcted female mice (male MI [0.7 ± 0.1 µg/protein] versus female MI [0.4 ± 0.04 μg/protein], *P* < 0.05; Figure 6G). No differences were observed in mitochondrial OCR (Basal, ATP-linked, FCCP-induced) of the vagal ganglia of sham male versus sham female mice (Supplemental Figure 4).

### 17β-Estradiol reduces cardiac parasympathetic dysfunction following MI in male mice.

**Figure 7:**
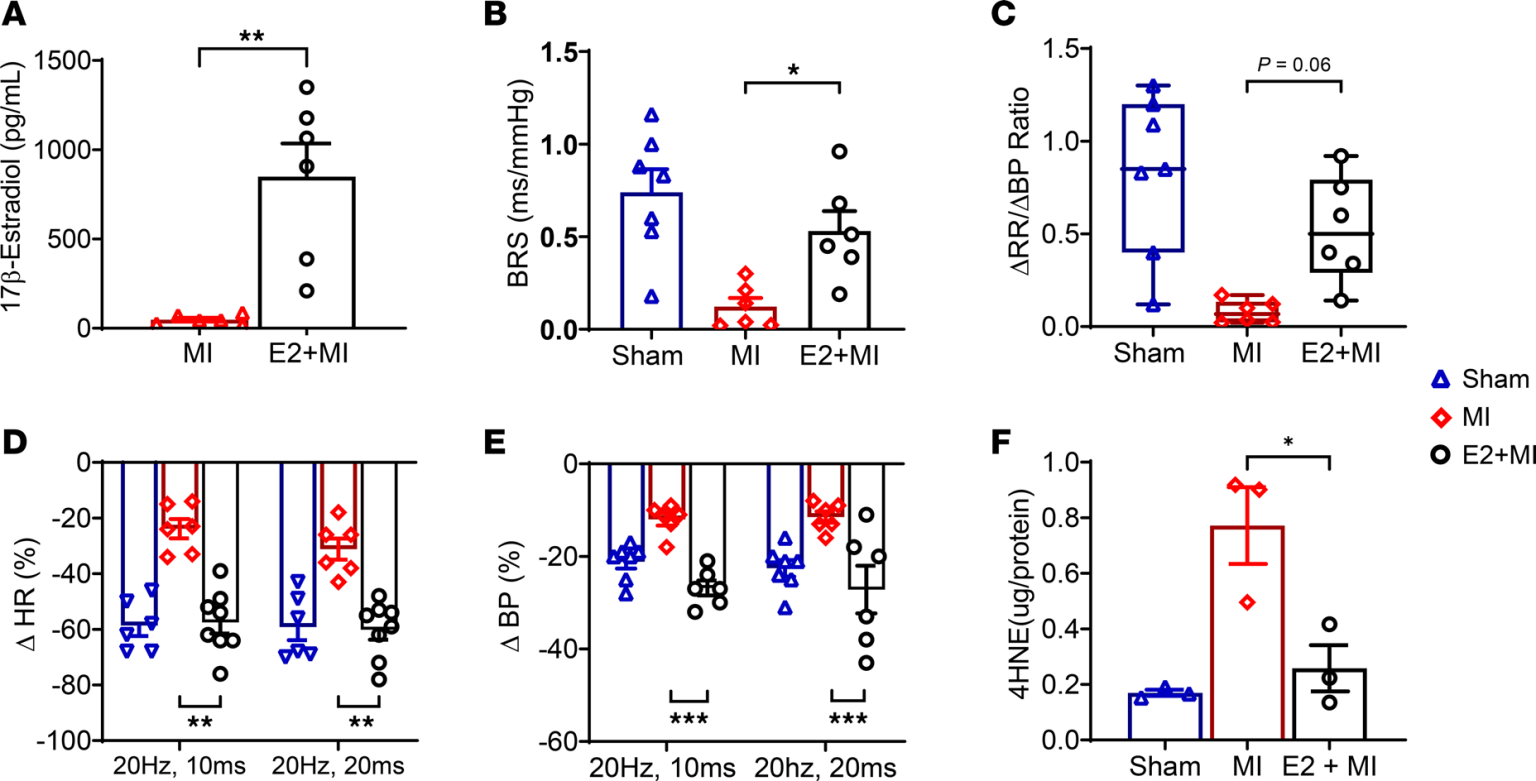
17β-Estradiol ameliorates cardiac parasympathetic dysfunction following MI in male mice. (**A**) Plasma E2 levels from E2 + MI animals were confirmed to be elevated versus MI-only animals (*P* < 0.01, *n* = 6 per group). (**B** and **C**) BRS improved (*P* < 0.05), and there was a trend for improvement in the ratio of ΔRR/ΔBP (*P* = 0.06) in E2 implanted infarcted (E2 + MI) versus MI-only male mice (*n* = 6 per group), while no significant differences were noted between sham (*n* = 7) and E2 + MI males. (**D**) E2 + MI males also demonstrated improved HR responses to optogenetic stimulation compared with MI-only animals (*P* < 0.01). No significant differences between E2 + MI versus sham males were noted. (**E**) Similar to HR, blood pressure responses in E2 + MI animals were restored with estradiol (*P* < 0.001 for E2 + MI versus MI-only males, *n* = 6 per group) and were similar to sham (*n* = 7) animals. (**F**) Vagal ganglia were isolated (*n* = 12 ganglia/sample per group, 3 independent samples), and 4-HNE levels were quantified. 4-HNE levels were significantly lower in E2 + MI versus infarcted males (*P* < 0.05) and were not statistically different from sham male mice. Data are shown as mean ± SEM. *P < 0.05, **P < 0.01, ***P < 0.001 for comparisons of E2 + MI versus MI. Unpaired Student’s *t* test was used for comparison of E2 levels. One-way ANOVA with the FDR corrected for by the Benjamini-Hochberg procedure was used for the comparison of MI versus E2 + MI versus sham groups. Data are shown as mean ± SEM.

To determine whether estradiol can underly the observed sex differences in autonomic remodeling after MI, autonomic and molecular studies were carried out in infarcted male mice treated with 17β-estradiol (E2) approximately 2 weeks prior to MI (E2 + MI). Plasma measurements confirmed higher estrogen levels in E2-implanted mice (MI [48 ± 10 pg/mL], E2 + MI [850 ± 151 pg/mL]; MI versus E2 + MI, *P* < 0.01, *n* = 6 per group; Figure 7A).

BRS significantly improved with E2 pretreatment (*P* < 0.05; Figure 7B), while there was a trend for improvement in ΔRR/ΔBP (Figure 7C). Furthermore, in response to vagal optogenetic stimulations, E2 + MI male mice demonstrated significantly better reflex HR responses versus MI-only animals and similar responses to sham male animals (20 Hz, 10 ms: sham [–59% ± 3%], MI [–24% ± 3%], E2 + MI [–58% ± 4%], MI versus E2 + MI, *P* < 0.0001; 20 Hz, 20 ms: sham [–59% ± 5%], MI [–31% ± 4%], E2 + MI [–60% ± 3%], MI versus E2 + MI, *P* < 0.01; Figure 7D). E2 + MI male mice also demonstrated improved BP responses to optical stimulation (Figure 7E). 4-HNE levels were increased in MI versus sham animals, while E2 + MI mice demonstrated significantly lower 4-HNE levels than infarcted animals (sham [0.17 ± 0.01 μg/protein], MI [0.77 ± 0.13 μg/protein], E2 ± MI [0.25 ± 0.06 μg/protein], MI versus E2 + MI, *P* < 0.05; Figure 7F).

## Discussion

The mechanisms behind vagal dysfunction after MI remain unknown. This study demonstrates that MI causes significant impairment of glutamatergic sensory vagal neurotransmission. Impairment of vagal afferent neurotransmission appears to be, at least in part, responsible for the efferent vagal dysfunction/reflexes observed after MI. In addition, glutamate levels were found to be reduced in the vagal ganglia of male mice after MI, and this reduction was associated with post-MI mitochondrial dysfunction and increased oxidative stress. Furthermore, significant sex differences in autonomic vagal remodeling after MI were observed in baroreflex function, vagal glutamatergic afferent signaling, mitochondrial function, and oxidative stress, with males demonstrating more dysfunction compared with females. Finally, this study demonstrates that treatment with estradiol reversed the dysfunction in vagal neurotransmission and neural oxidative stress in males, providing direct data for the role of estradiol in vagal remodeling.

### Vagal afferent and efferent function after MI.

A significant highway for afferent and efferent communication between the brain and the heart is the vagus nerve, which innervates the atria, ventricles, and the conduction system (12, 34). Neurons in the inferior vagal ganglia (nodose ganglia) sense the chemical and mechanical milieu of the heart and transmit these signals to the brain stem, modulating autonomic efferent tone (12). Myocardial injury and infarction cause scar formation, leading to both pathological cardiac and concomitant neural remodeling (14). The resulting sympathoexcitation has been the subject of multiple studies, and sympathetic blockade remains an important therapeutic strategy for heart failure and ventricular arrhythmias (14). Although vagal dysfunction is known to increase both the risk of VT/VF and the progression of heart failure (15–18), the mechanisms underlying this dysfunction, which manifests as decreased efferent vagal tone, remain unclear. After chronic MI, the firing patterns of the postganglionic parasympathetic neurons in fat pads of the heart demonstrate changes consistent with decreased central vagal inputs (35), while acetylcholine levels in the heart remain preserved (35). A study in guinea pigs reported cholinergic dysfunction in postganglionic cardiac parasympathetic neurons 3 days after MI, due to impairment of the nitric-oxide cGMP pathway, which was mitigated with adenoviral neuronal nitric oxide synthase delivery (36). However, studies that have performed electrical vagal nerve stimulations, both with the nerve intact and after bilateral vagotomy with distal-end stimulation, have reported greater cardiovascular effects in chronically infarcted compared with sham animals, suggesting that efferent cardiac vagal pathways remain predominantly preserved or can be overdriven by electrical stimulation after MI (35, 37). Regardless, in animal models of heart failure, augmenting the efferent vagal drive via vagal nerve stimulation increases the ventricular refractory period and decreases ventricular dispersion of repolarization, reducing VT/VF inducibility (35, 38); this suggests that enhanced efferent vagal tone can be antiarrhythmic. In this study, we hypothesized that decreased efferent vagal tone after MI may also be related to afferent dysfunction. Our results indicate that MI is associated with vagal glutamatergic sensory dysfunction, which can, in turn, lead to the observed decreased central efferent vagal drive after MI and interfere with vagal reflexes, such as baroreflex function.

Notably, the magnitude of impairment in vagal glutamatergic afferent signaling was greater in male compared with female mice after MI. In line with our findings, human HR variability data indicate that middle-aged women prior to menopause (40–46 years old) exhibit greater vagal tone and decreased sympathetic tone, compared with age-matched men (39, 40). The greater vagal tone in women may have antiarrhythmic effects. It has been reported that the risk of the first and subsequent episodes of VT/VF and ICD shocks are lower in women than in men with cardiomyopathy and ventricular dysfunction (7). Women are also less likely to have inducible ventricular arrhythmias in the setting of ischemic cardiomyopathy and prior MI, and they are less likely to have ICD shocks and appropriate ICD therapies for spontaneous VT/VF, even in the setting of significant structural heart disease (4, 7, 41). Finally, decreased rates of age-adjusted SCD are noted in women (42), but this difference diminishes after menopause, so that at the age of 65 or older, the incidence of SCD is similar in men and women (43). Notably, the differences in HR variability parameters have also been shown diminish after menopause, especially after the age of 60 (39, 40), while estrogen replacement has been shown to improve vagal tone. Frequency domain analyses have shown that estrogen replacement therapy increases high frequency power (HF) and reduces low frequency power (LF) to HF ratio, suggesting that estrogen replacement improves vagal tone (40). Estrogen has also been shown to improve baroreflex function in postmenopausal women (44). Administration of estrogen increases BRS in ovariectomized female rats (45), and estrogen has also been shown to protect against oxidative stress and mitochondrial dysfunction in the myocardium and the brain (46, 47). Hence, estrogen may underlie the observed sex differences in autonomic remodeling. Our data suggest that parasympathetic dysfunction and oxidative stress in the vagal ganglia are ameliorated by estradiol, suggesting that estrogen protects against vagal remodeling after MI, reducing the risk of VT/VF and SCD.

### Glutamate production and sex differences.

Glutamate, the most abundant excitatory neurotransmitter in the central and peripheral nervous systems, is critical for sensory autonomic neurotransmission (48). In our study, reductions in vagal glutamate levels were significantly greater in infarcted versus sham male mice, whereas female infarcted mice had greater glutamate levels than male infarcted mice. Glutamate is synthesized in 2 different ways: (a) glutamine from glial cells is taken up by neurons through an excitatory amino acid transporter and then converted to glutamate by the mitochondrial enzyme, glutaminase, and (b) glutamate is formed from the citric acid cycle intermediate, α-ketoglutarate by glutamate dehydrogenase (49). Therefore, glutamate production is dependent, in part, on a normal citric acid cycle and mitochondrial function. In our study, basal oxygen consumption levels and ATP-linked oxygen consumption levels were significantly lower in the vagal ganglia obtained from infarcted males compared with infarcted females, suggesting that mitochondrial dysfunction may be responsible, at least in part, for the impaired glutamate-mediate neurotransmission.

It’s possible that increased myocardial oxidative stress after MI induces oxidative injury in vagal afferent neurons by affecting sensory nerves of the vagal ganglia, whose axons reside in the myocardium. It is also possible that the initial excitatory neuronal activity associated with acute MI causes excitotoxicity, which results in mitochondrial dysfunction and subsequent reduction in glutamate levels. Free radicals are generated from the heart during MI, and this increased oxidative stress can adversely affect the various tissues within an organ (50). In this study, we also investigated whether the mechanism behind mitochondrial dysfunction may be related to increased oxidative stress after MI and whether the sex differences in this dysfunction could be mediated by E2, given that estrogen has been reported to protect against oxidative stress in the myocardium (51). Levels of 4-HNE, a marker for oxidative stress, were increased in the vagal ganglia of male compared with female infarcted mice. Notably, pretreatment with E2 reduced 4-HNE levels after chronic MI, suggesting that the sex differences in oxidative stress could be due in part to differences in estrogen’s action or availability.

In summary, this study demonstrates that MI leads to impaired vagal sensory function that is, at least in part, due to impaired glutamatergic sensory neurotransmission and is associated with underlying decreased glutamate production, mitochondrial dysfunction, and elevated oxidative stress. This sensory dysfunction can be responsible for the decreased reflex efferent vagal drive that has been observed across multiple species after MI. Finally, these data point to the existence of potentially novel sex differences in parasympathetic remodeling after MI that is modulated by E2.

### Limitations.

Vagal BRS testing and assessment of vagal glutamatergic afferent function were performed under anesthesia, which can blunt autonomic reflexes. However, similar levels of anesthesia were used for all male and female MI, sham, and healthy animals. We did not specifically test the effects of efferent vagal stimulation. Cholinergic dysfunction has been noted in the postganglionic neurons of infarcted guinea pigs (36), though activation of efferent vagal fibers by electrical stimulation has been demonstrated to lead to similar or even greater cardiovascular responses in infarcted versus normal animals and does not explain the reduction in central vagal drive observed after chronic MI (35, 37). Although this study focused on left vagal sensory neurotransmission for optogenetic studies (as the right side was often used for placement of the Millar pressure transducer for BP recordings), ventricular efferent vagal responses and histological remodeling of the nodose ganglia after MI have been reported to be similar, regardless of location of infarction, without evidence of unique lateral effects (38, 52); furthermore, ventricular sensory neurons have been shown to be present in bilateral nodose ganglia (53). Optogenetic stimulation and molecular analyses of Vglut2 neurons do not differentiate between organ-specific neuronal subtypes. The striking changes observed in responses to optogenetic stimulation of these vagal afferents, however, may suggest that inflammation/injury to one organ may potentially affect autonomic tone to other organs, as has also been observed clinically (i.e., inflammatory bowel disease is associated with reduced HR variability and MI with gastrointestinal dysfunction/gastroparesis) (54, 55). While Vglut2 has been shown to be predominantly selective for afferent vagal fibers and neurons (56), a very small number of efferent preganglionic neurons can also express Vglut2, and optogenetic stimulation of these fibers cannot be excluded. Vagal dysfunction in this study was associated with significant mitochondrial dysfunction in the vagal ganglia after MI; however, assessments to clearly demonstrate a cause-effect relationship — for example, by restoring mitochondrial function to demonstrate resolution of vagal dysfunction — were not performed and remain subject of future studies. Our study evaluated sex differences to vagal afferent activation (including with optical stimulation and baroreflex testing). Sex differences in tonic vagal tone after MI remain an important topic of investigation for future studies. In addition, in this study, E2 levels were measured only in male mice (infarcted and those implanted with E2 pellets), and comparable measurements were not performed in females. The levels measured were above physiological levels reported in female mice in other studies (57–59), though measurements can vary by the methodology used. Other studies have also suggested that E2 supplementation using methods such as s.c. silastic capsules or via oral administration of nut-cream Nutella may be superior in producing more physiological E2 levels compared with the commercially available slow-release pellets used in this study (57, 58). Finally, systemic administration of E2 may also improve vagal reflexes beyond a nodose-dependent mechanism.

## Methods

### Sex as a biological variable.

Sex as a biological variable was examined in this study, and both male and female mice were used.

### Animal use and ethical approval.

The study was powered based on detecting optogenetic differences in HR responses in male infarcted versus sham mice at α = 0.05 and β = 0.85 as well as anticipated 10%–20% differences in responses between male versus female infarcted mice. Vglut2-ires-cre (The Jackson Laboratory, 028863), Ai32 (Jackson Labs, 024109) and C57BL/6J (The Jackson Laboratory, 000664) were used. Mice were housed in individual cages with ad libitum access to water, exposed to 12-hour light/dark cycles at 21°C–23°C, and were fed a standard diet. The Vglut2-ChR2-EYFP mice were generated by crossing the Vglut2-ires-Cre mice with ChR2-EYFP mice.

### Creation of myocardial infarcts.

Mice (12–14 weeks of age) underwent creation of myocardial infarcts. One hour prior to surgery, a nonsteroidal antiinflammatory drug, carprofen (5 mg/kg, s.c.), and an analgesic drug, buprenorphine (0.1 mg/kg, s.c.), were administered. Mice were then placed under general anesthesia (inhaled isoflurane, 5% for induction and 1%–3% for maintenance), intubated, and placed on a heated surgical board/pad. An incision was made in the fourth intercostal space, and the heart was exposed. The LAD coronary artery was permanently ligated 2 mm from its origin with an 8–0 proline suture. MI was confirmed by blanching of myocardial tissue as well as by ST-segment changes observed on the surface ECG. The thorax was closed in layers. Sham surgeries were performed as described above, except that coronary artery ligation was not performed. Throughout the procedure, body temperature was monitored with a rectal probe and maintained by using a circulating water heating pad.

### Implantation of E2 pellets.

In male mice (*n* = 25), 0.5 mg sterile E2 pellets (Innovative Research of America) were implanted 2 weeks prior to MI procedures (E2 + MI group). The dose of E2 pellets was selected based on a prior study evaluating sex differences in the setting of a heart failure with preserved ejection fraction mouse model (60). Briefly, mice were placed under anesthesia, and a small incision was made on the lateral aspect of the neck; a pocket was created on the dorsal aspect approximately 1–2 cm beyond the incision site, a pellet was placed with the pocket, and the incision sutured closed. Blood was collected at terminal procedures, and plasma E2 was measured by 17β-Estradiol ELISA kit in a subset of male infarcted mice, per the manufacturer’s protocol (Abcam, ab108667).

### BRS testing.

BRS was performed in sham males (*n* = 7), infarcted males (*n* = 6), female sham (*n* = 6), infarcted females (*n* = 8), and infarcted males with E2 implants (*n* = 6). Mice were intubated and placed under general anesthesia (1%–2% inhaled isoflurane). Three ECG leads were inserted s.c. for HR/RR interval recordings. A midline incision was made in the neck and the right carotid artery was exposed. A 1F Millar catheter (Millar Medical) was inserted into the lumen of the artery and advanced until a clean arterial waveform was obtained for continuous BP recordings. Phenylephrine (4 mg/kg) was injected through a branch of the left jugular vein, and continuous ECG and BP recordings were obtained. BRS was calculated as the slope of the beat-to-beat change in RR interval versus the change in systolic BP (15, 17, 26). In addition, the absolute change in RR interval with respect to the absolute change in mean BP was calculated for each animal (61).

### In vivo optogenetic vagal stimulation.

Optogenetic studies were performed in healthy ChR2-EYFP mice (*n* = 4), Vglut2-ires-Cre mice (*n* = 5), and Vglut2-ChR2-EYFP mice (*n* = 3) as well as in Vglut2-ChR2-EYFP sham males (*n* = 6–7), infarcted males (*n* = 6), infarcted female (*n* = 6–7), E2 + MI males (*n* = 8) to assess hemodynamic responses to activation of afferent glutamatergic neurons. Optogenetic studies were performed 2–3 weeks after the creation of MI or sham procedures. Animals were anesthetized with isoflurane (5% induction, maintenance at 1%–3%), intubated, and mechanically ventilated. Core body temperature was maintained at 37°C. ECG recordings were obtained as above. The left cervical vagus nerve was exposed, following a midline neck incision. A laser-coupled optical fiber was positioned just above the nerve (position was confirmed under the microscope). Optical stimulation was performed via a 473 nm DPSS laser system (Optoengine LLC) coupled to a constant current stimulator (Grass, PSIU6 and Model S88X, output 3V) initially at 10, 20, and 30 Hz in healthy animals and then at 20 Hz, 10 ms, and 20 Hz, 20 ms, in infarcted and sham animals in vivo. Measured optical intensity was 67 mW for 20 Hz, 10 ms, and 108.8 mW for 20 Hz, 20 ms, stimulation. All stimulations were performed for 5 seconds, with at least a 5-minute interval allowed in between stimulations for hemodynamic parameters to return to baseline values. The mean BP responses to optical stimulation, calculated as systolic BP plus 2 times diastolic BP divided by 3, were assessed in 7 sham males, 6 infarcted males, 7 infarcted females, and 6 E2 + MI using a pressure transducing catheter (1F Millar Catheter, Millar Instruments) inserted into the right carotid artery at baseline (~3–5 seconds prior to stimulation) and during stimulation.

### Glutamate measurements.

Briefly, vagal ganglia were collected from sham male, sham female, infarcted male, and infarcted female mice, snap frozen, and stored at –80°C. Vagal ganglia were pooled from 3 independent samples of infarcted males (*n* = 18 nodose ganglia pooled from 9 animals, per group, 3 independent samples). Glutamate levels were measured using ELISA, based on the manufacturer’s protocol (Abnova, KA1909). Briefly, ganglia were homogenized from pooled samples. After extraction and derivatization, 25 μL of samples or standard were plated on a 96-well plate and 50 μL glutamate antiserum was added. Samples were then incubated for 15–24 hours at 4°C. After washing the buffer 3 times, 100 μL of enzyme conjugate was added, and samples were then incubated for 30 minutes at room temperature. The contents were then washed 3 times, and 100 μL substrate was added to each well and incubated for 30 minutes. Horseradish peroxidase activities were measured using an ELISA plate reader after adding 100 μL of stop solution.

### Vglut2 measurements.

Vglut2 levels were quantified in the vagal ganglia of experimental groups (*n* = 6 animal per group [i.e., 12 ganglia per group], 3 independent samples). Tissue extracts were plated onto a 96-well plate, kept at 4°C overnight, and washed 3 times with phosphate-buffered saline (pH 7.4), containing 0.05% Tween 20, blocked with 3% BSA in phosphate-buffered saline (100 μL/well), and kept at 37°C for 1 hour. The samples were washed, and 100 μL of rabbit Vglut2 primary antibody (1:1000, Abcam, ab216463) was added. The samples were then incubated at 37°C for 1 hour, followed by washes with phosphate-buffered saline and Tween. Anti–rabbit IgG (1:5,000, Thermo Fisher Scientific, 31460) conjugated with horseradish peroxidase was then added. The plates were then incubated at 37°C for 1 hour and washed again. The activity of bound horseradish peroxidase was measured by the addition of 50 μL of substrate. The reaction was then arrested by stop solution. Samples were analyzed using an ELISA reader at 450 nm (BMG-Labtech)

### Assessment of mitochondrial function in the vagal ganglia.

Cellular metabolic rates were measured using an XF24 Extracellular Flux Analyzer (Seahorse Bioscience). Vagal ganglia were isolated from infarcted males (*n* = 6 ganglia), infarcted females (*n* = 6 ganglia), sham males (*n* = 8 ganglia), and sham females (*n* = 8 ganglia) and placed in V7 plates (Seahorse Bioscience). Immediately before measurements, the ganglia were washed with unbuffered complete DMEM, as per manufacturer protocol (62). Mixing, waiting, and measurement times were 0.5, 2, and 3 minutes, respectively (an extra 0.5 minutes was added after each injection). Rotenone was used for inhibiting mitochondrial respiratory complex I, and antimycin was used for inhibition of mitochondrial respiratory complex III. FCCP was used for measuring maximum respiration capacity. Mitochondrial respiratory activities were normalized based on protein concentrations. Protein concentration estimates were carried out using the BCA method, as described in the manufacturer’s protocol (Thermo Fisher Scientific). Briefly, after assessment of mitochondrial function in the vagal ganglia, the medium was removed from the V7 plate, and the nodose ganglia were sliced into small pieces with Vannas spring scissors. Then, 10 μL protein lysate buffer was added, and samples were frozen and thawed 3 times. Protein lysate was mixed with reagents and incubated for 30 minutes; absorbance was measured at 562 nm using a plate reader (BMG Labtech).

### Lipid peroxidation (LPO) measurements.

Vagal ganglia were isolated from experimental groups (*n* = 6 animals per group [i.e., 12 ganglia per group], 3 independent samples). LPO levels were measured in the lysate, per the manufacturer’s protocol, using a 4-HNE ELISA Kit (Abcam, ab238538). Briefly, 50 μL samples were loaded into 4-HNE conjugate–coated wells and incubated at room temperature for 30 minutes on an orbital shaker. Samples were washed 3 times with washing buffer, anti–4-HNE antibody was added to each well, and samples were incubated at room temperature for 1 hour on an orbital shaker. Samples were again washed 3 times, a 100 μL of the diluted secondary antibody-HRP conjugate was added, and samples were incubated for 1 hour at room temperature on an orbital shaker. After washing 3 times with washing buffer, 100 μL of substrate solution was added, and samples were again incubated for 20 minutes. Standards were performed in parallel. Absorbance was measured at 450 nm after adding the stop solution.

### Statistics.

Data are reported as mean ± SEM. After confirmation of Gaussian distribution, unpaired, 2-tailed Student’s *t* test was used to compare parameters between groups (i.e., sham versus MI and females versus males) and 2-tailed paired Student’s *t* test was used for within-group comparisons. Comparison of parameters across 3 groups were performed using a 1-way repeated-measures ANOVA, with the FDR corrected for by the Benjamini-Hochberg procedure. Statistical analyses were performed using GraphPad Prism software (version 8.4.3). Data points were excluded from optogenetic studies in cases where the Millar catheter had been displaced from its original position or when unclear, noisy, or weak waveforms were noted due to clotting around the catheter.

### Study approval.

This study conformed to the NIH Guide for the Care and Use of Laboratory Animals. The protocol was approved by the UCLA IACUC.

### Data availability.

Values for all the data points presented in the figures and manuscript can be found in the Supporting Data Values file.

## Author contributions

AD, AJL, EDA, ALH, JDT, and MV were involved in the design of the experiments and the development of protocols. KW, ZAL, AD, KS, and JDT contributed to performing the experiments. AD, ZAL, and ME processed tissue and performed assays. AD, KW, ZAL, EDA, ALH, AJL, and MV were involved in data analysis and/or interpretation of the data. AD and MV drafted the manuscript. All coauthors contributed to the final manuscript.

## Supplementary Material

Supplemental data

Supporting data values

## Version 1 06/17/2024

In-Press Preview

## Version 2 07/22/2024

Electronic publication

## Version 3 11/27/2024

The supporting grant number was added
