# Supplementary material for: Myocardial infarction causes sex-dependent dysfunction in vagal sensory glutamatergic neurotransmission that is mitigated by 17β-estradiol
Source: JCI Insight. 2024 Jul 22;9(14):e181042. doi: 10.1172/jci.insight.181042 (PMC11383359; doi:10.1172/jci.insight.181042)
Supplement: Supplemental data [file jciinsight-9-181042-s008.pdf]

Supplemental Figure 1.

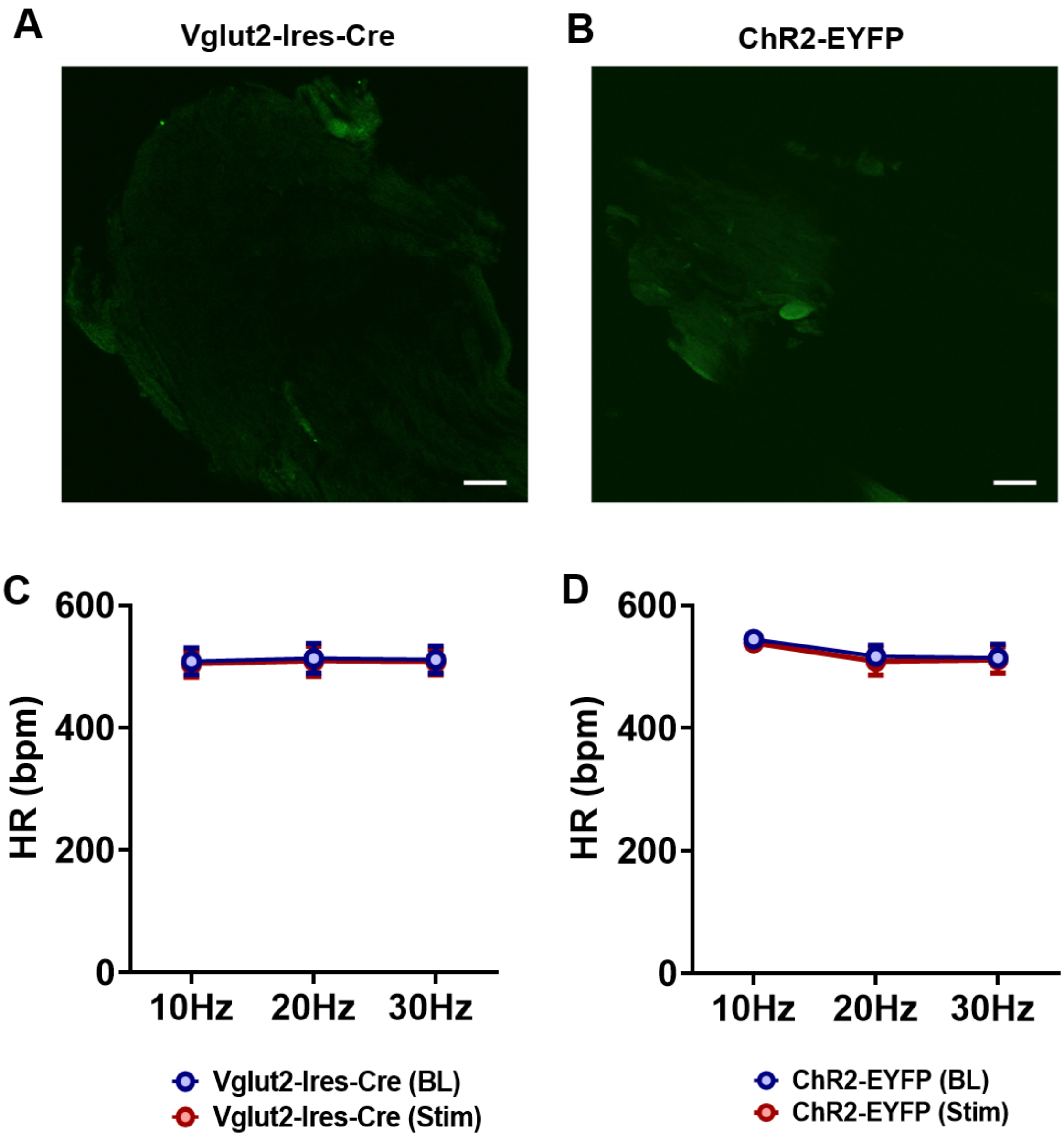

Supplemental Figure 1. (A-B) Nodose ganglia were isolated from Vglut2-Ires-Cre and ChR2-EYFP mice and checked for the presence of EYFP. EYFP is absent in the nodose ganglia isolated from Vglut2-Ires-Cre and ChR2-EYFP. **(C-D)** The left vagus nerve was isolated in Vglut2-Ires-Cre and ChR2-EYFP mice for optogenetic stimulations. In-vivo responses to optogenetic stimulation in Vglut2-Ires-Cre (n=5) and

ChR2-EYFP mice (n = 4) are shown. No heart rate response was observed in Vglut2-Ires-Cre and ChR2-EYFP mice following optogenetic stimulation.

Supplemental Figure 2.

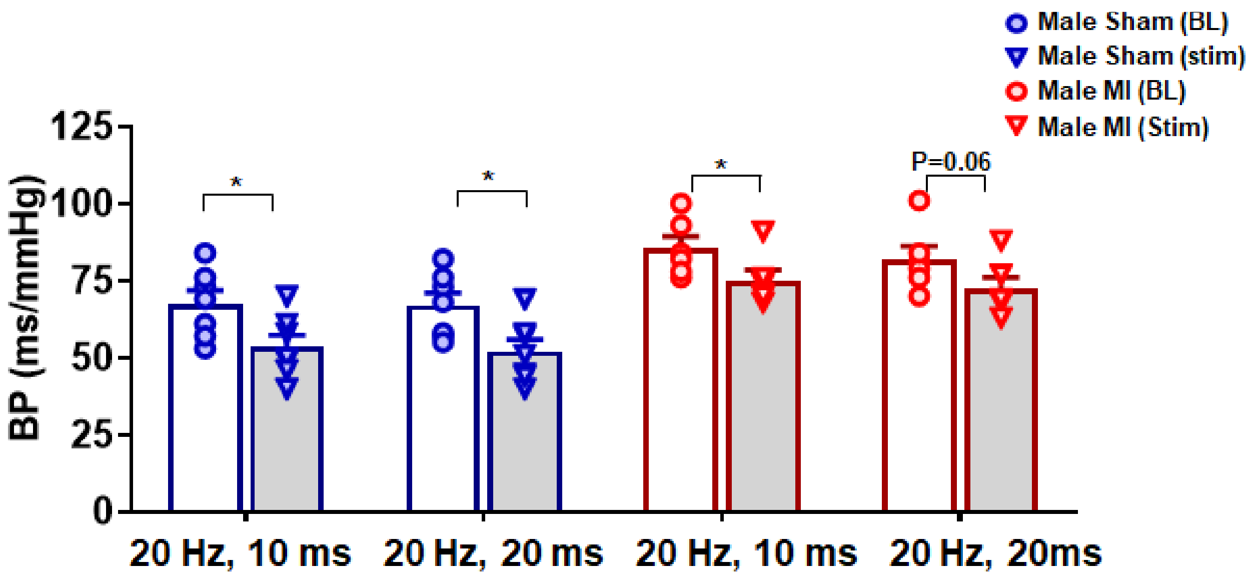

Supplemental Figure 2. Blood pressure responses to optogenetic stimulation of vagal sensory neurons in sham and infarcted male mice in-vivo. Two to three weeks post-MI, in-vivo optogenetic stimulation (20 Hz, 10 ms, and 20 Hz, 20 ms) of the left vagus nerve was performed, and mean blood pressure (BP) responses (n = 7 sham and n = 6 MI animals) were measured. Blood pressure was reduced in response to optogenetic stimulation in male Vglut2-ChR2-EYFP sham and infarcted mice compared to baseline. Paired Student's t-test was performed for comparison of baseline to stimulation parameters. Data are shown as mean ± SEM. **P* < 0.05, ***P* < 0.01, ***P* < 0.001. BL = baseline prior to stimulation, Stim = during optical stimulation.

Supplemental Figure 3.

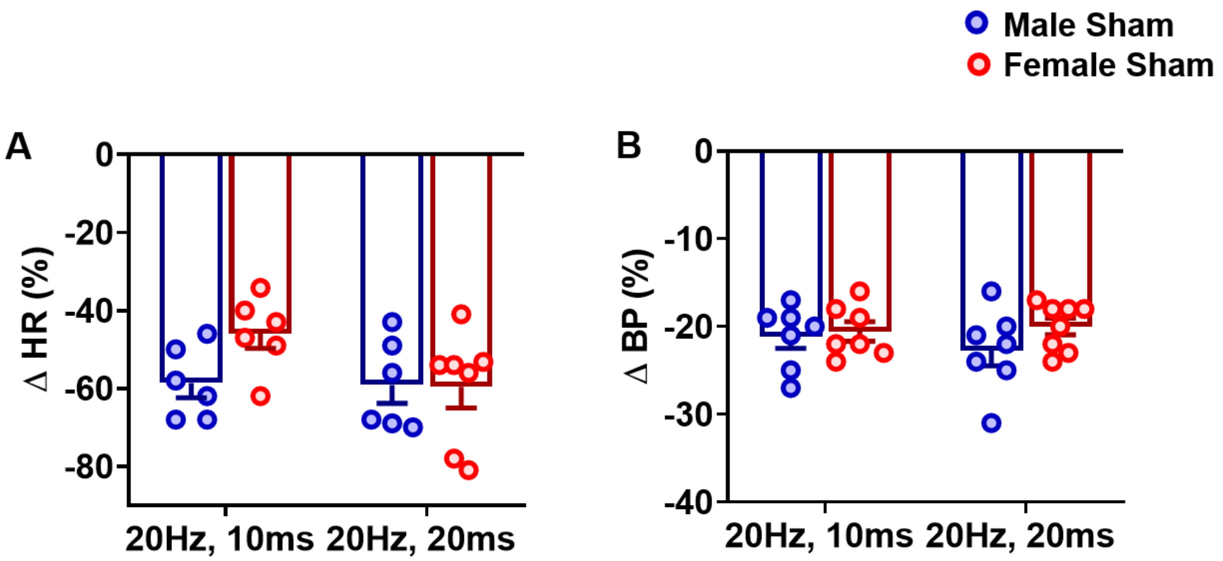

Supplemental Figure 3. Optogenetic stimulation of vagal sensory neurons in female and male sham mice. *In-vivo* optogenetic stimulation of the left vagus nerve was performed two to three weeks post-sham procedures (20 Hz, 10 ms, and 20 Hz, 20 ms) in male (n = 6-7) and female (n= 6-8) Vglut2-ChR2-EYFP sham mice. **(A)** HR and **(B)** blood pressure (BP) responses were evaluated. Neither HR nor blood pressure responses were different in sham male vs. sham female mice. Unpaired Student's t-test was used for inter-group comparisons. Data is shown as mean ±SEM.

Supplemental Figure 4.

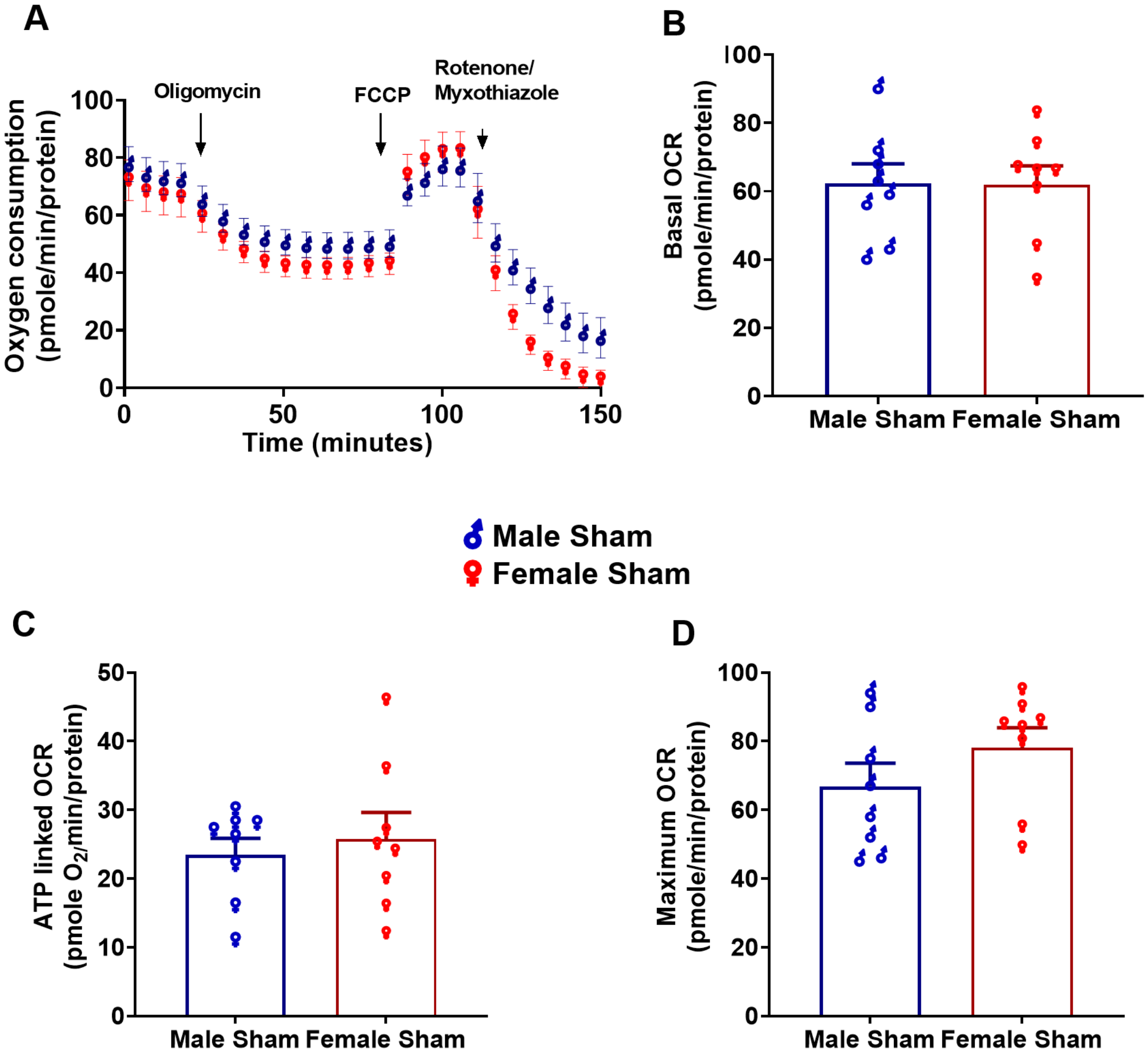

Supplemental Figure 4. Assessment of mitochondrial function in the vagal ganglia of male sham and female sham mice. (A) Oxygen consumption was assessed in the vagal ganglia isolated from male and female Vglut2-ChR2-EYFP sham mice (n = 8 ganglia for male sham and n = 8 ganglia for female sham). No difference in **(B)** basal, **(C)** ATP-linked, or **(D)** FCCP-induced oxygen consumption rates was found in

male sham compared to female sham mice. Unpaired Student's t-test was used for intergroup comparisons. Data shown as mean ± SEM.
